# Prenatal bisphenol A exposure reprograms SF1-lactylation pathways to promote endometriosis susceptibility

**DOI:** 10.1016/j.isci.2026.115608

**Published:** 2026-04-04

**Authors:** Xiaohan Liu, Yanxia Fu, Liuxuan Huang, Donghan Li, Paul Yao, Liqin Zeng

**Affiliations:** 1 Department of Gynecology, The Eighth Affiliated Hospital, Sun Yat-Sen University, Shenzhen 518033, P.R. China

**Keywords:** therapy, molecular interaction, developmental biology

## Abstract

Endometriosis is an estrogen-dependent disorder influenced by dysregulated steroidogenesis, oxidative stress, and inflammation. Bisphenol A (BPA), a common endocrine disruptor, has been associated with reproductive dysfunction, but its developmental impacts are unclear. We investigated whether prenatal BPA exposure induces lasting endocrine and epigenetic reprogramming that increases endometriosis susceptibility. In human endometrial stromal cells, BPA elevated histone lactylation, SF1, and CYP19A1 expression, oxidative stress, mitochondrial impairment, and inflammatory signaling. In a prenatal BPA mouse model, adult female offspring exhibited increased endometriosis-like lesions and reduced fertility. Pharmacologic inhibition of glycolytic lactate production or SF1 signaling (NaOx, AC45594) partially reversed these outcomes. These findings show that prenatal BPA drives persistent SF1-linked estrogenic activation and lactylation, promoting endometriosis risk and suggesting targeted therapeutic strategies.

## Highlights

• Prenatal BPA exposure induces persistent endocrine and epigenetic reprogramming
• BPA enhances histone lactylation and activates SF1-mediated steroidogenesis
• BPA impairs mitochondrial function and promotes oxidative and inflammatory signaling
• Prenatal BPA exposure leads to endometriosis-like lesions and subfertility in offspring

## Graphical abstract

**Figure:**
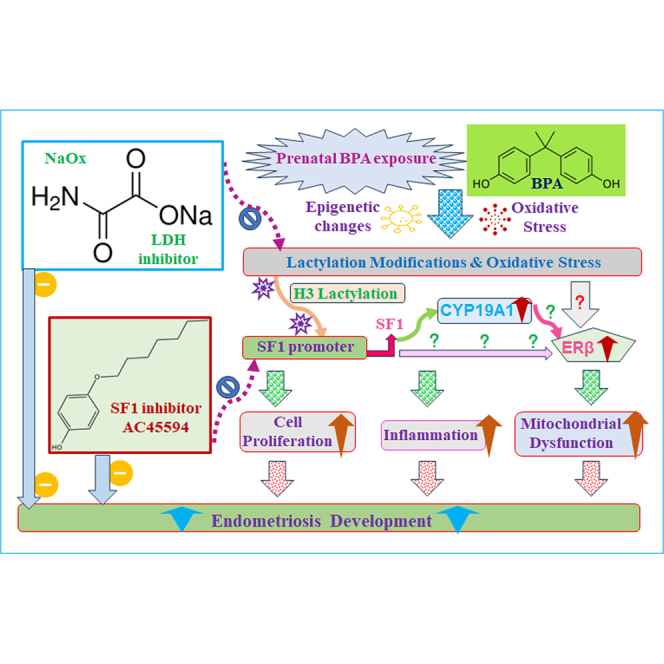

## Introduction

Endometriosis is a chronic, estrogen-dependent disorder characterized by the ectopic growth of endometrium-like tissue, which frequently results in pelvic pain and reduced fertility in ∼10% of reproductive-age women.^1^^,^^2^^,^^3^ Estrogen is central to the pathogenesis of endometriosis by promoting proliferation and survival of ectopic endometrial cells. In particular, estrogen receptor beta (ERβ) is overexpressed in endometriotic lesions, where it contributes to immune evasion, chronic inflammation, and resistance to apoptosis.^4^^,^^5^ Targeting ERβ has emerged as a promising therapeutic strategy, with selective antagonists demonstrating efficacy in reducing lesion size and inflammation in preclinical models.^6^^,^^7^^,^^8^^,^^9^ Additionally, epigenetic modifications and altered estrogen metabolism may contribute to disease persistence and recurrence,^10^ highlighting the need to better understand the molecular pathways driving endometriosis.

Bisphenol A (BPA), a commonly encountered endocrine disruptor, plays a role in the onset and advancement of endometriosis. BPA functions as an estrogen mimic by engaging estrogen receptor pathways, thereby promoting proliferation and inflammation in ectopic endometrial tissues.^11^ Rodent studies have demonstrated that BPA exposure increases both the size and number of endometriotic lesions.^12^ Notably, prenatal BPA exposure may predispose female offspring to endometriosis-like phenotypes by disrupting uterine development and immune function during fetal life.^13^^,^^14^ Such early-life exposure can cause long-lasting epigenetic changes and aberrant estrogen signaling, supporting the developmental origins hypothesis of endometriosis and raising concerns about BPA’s role in increasing disease susceptibility.^15^^,^^16^

Steroidogenic factor-1 (SF1; NR5A1), a nuclear receptor critical for steroid hormone biosynthesis, is ectopically expressed in endometriotic lesions but not in the normal endometrium. Its aberrant expression drives local estrogen production by upregulating CYP19A1 (aromatase) and other steroidogenic enzymes, thus sustaining lesion growth and inflammation in human tissues.^17^^,^^18^ SF1 also modulates prostaglandin signaling and immune responses in the ectopic microenvironment.^19^ Epigenetic mechanisms, such as promoter hypomethylation, may underlie its abnormal activation in endometriosis.^17^ Inhibition of SF1 was found to suppress lesion size along with local estrogen levels, making it an attractive therapeutic target.^20^

Histone lactylation, a recently identified epigenetic modification derived from lactate, has been identified as a crucial modulator of gene expression in inflammation and tissue remodeling. In the context of endometriosis, elevated local lactate levels may enhance histone lactylation, thereby promoting pro-inflammatory gene transcription and fibrotic changes in ectopic lesions.^21^ Lactate-induced histone lactylation has also been shown to activate macrophages and drive M2 polarization, further contributing to the inflammatory microenvironment.^22^ Moreover, aberrant histone lactylation may influence the transcription of estrogen-related genes, linking cellular metabolism with hormonal signaling in endometriosis.^23^^,^^24^ Thus, targeting histone lactylation represents a potential therapeutic strategy.

In this study, we aim to investigate whether prenatal BPA exposure promotes endometriosis-like phenotypes in female mouse offspring and whether this effect is mediated through histone lactylation and upregulation of SF1. We utilized a BPA dose (50 μg/kg/day) that has been well characterized in the literature as sufficient to induce endocrine and reproductive alterations without eliciting overt maternal toxicity. This intermediate exposure level provides a valuable window for detecting subtle but biologically meaningful perturbations in hormone-regulated reproductive processes. Similar doses have been shown to disrupt ovarian folliculogenesis, uterine gene expression, and steroid receptor signaling in rodents.^25^^,^^26^^,^^27^ Consistent with these reports, our findings indicate that prenatal exposure at this dose provokes measurable reproductive deficits and endometriotic lesion formation, highlighting its translational relevance to human low-dose environmental exposure scenarios. Employing both cell culture and animal models, we will establish a causal relationship between histone lactylation and SF1 expression. Furthermore, we will assess the therapeutic potential of blocking lactylation and SF1 signaling using the lactate dehydrogenase (LDH) inhibitor sodium oxamate (NaOx) and the SF1 inhibitor AC45594. Lastly, we will evaluate whether prenatal BPA exposure compromises fertility in female offspring. Collectively, these studies will provide mechanistic insights into how prenatal BPA exposure epigenetically enhances endometriosis in female offspring and impacts their reproductive health.

## Results

### SF1 and related genes are upregulated by BPA-induced histone lactylation on the SF1 promoter

**Figure 1:**
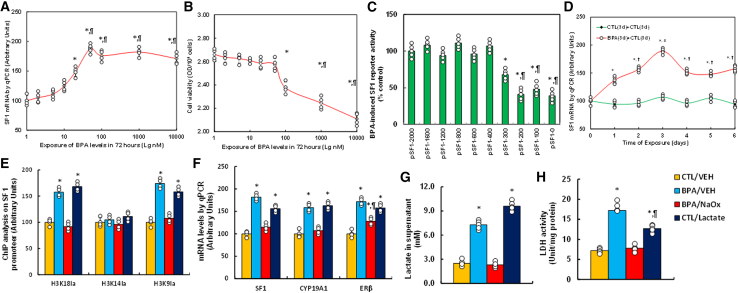
BPA exposure induces SF1 upregulation via histone lactylation at the SF1 promoter (A and B) Human endometrial stromal cells (HESCs) were treated with various BPA concentrations for 72 h. (A) SF1 mRNA levels (*n* = 4); ∗, *p* < 0.05 vs. 1 nM group; ¶, *p* < 0.05 vs. 20 nM group. (B) Cell viability (*n* = 5); ∗, *p* < 0.05 vs. 1 nM group; ¶, *p* < 0.05 vs. 100 nM group. (C) Luciferase assay in immortalized HESCs transfected with full-length or truncated SF1 promoter constructs (*n* = 5); ∗, *p* < 0.05 vs. pSF1-2000;¶, *p* < 0.05 vs. pSF1-300. (D) HESCs were treated with 50 nM BPA for 3 days, followed by chemical removal and further culture in fresh medium. SF1 mRNA was analyzed at indicated time points (*n* = 4); ∗, *p* < 0.05 vs. day 0; ¶, vs. day 1; #, vs. day 2. (E–H) HESCs treated with control (CTL/VEH), BPA (50 nM) alone (BPA/VEH), BPA + NaOx (20 mM), or BPA + lactate (10 mM) for 72 h. (E) ChIP analysis of the SF1 promoter (*n* = 4). (F) mRNA levels (*n* = 4). (G) Lactate levels (*n* = 5). (H) LDH activity (*n* = 5). *n* means number of independent repeats. ∗, *p* < 0.05 vs. CTL/VEH; ¶, *p* < 0.05 vs. BPA/VEH. Data are presented as mean ± SD.

HESCs were subjected to different levels of BPA exposure (1–10,000 nM) for 72 h, with BPA dissolved in ethanol (final concentration 0.1% in all groups). SF1 mRNA expression significantly increased at 20 nM BPA, peaking at 50 nM (Figure 1A), while cytotoxicity was observed at 100 nM via MTT assay (Figure 1B). To identify BPA-induced histone lactylation and transcriptional activation sites within the SF1 promoter region, progressively truncated 5′ constructs were transfected into immortalized HESC. Luciferase reporter assays showed reduced activity at the −300 bp construct (pSF1-300) compared to −2,000 bp (pSF1-2000), and further reduction at −200 bp (pSF1-200), indicating the BPA-responsive region lies between −300 and 0 bp (Figure 1C). To assess persistence, BPA was removed after 3 days of treatment. SF1 mRNA peaked on day 3, declined by day 4, but remained elevated through day 6, suggesting lasting epigenetic effects (Figure 1D). ChIP-qPCR revealed that BPA significantly increased histone lactylation at H3K18la and H3K9la, but not H3K14la (Figure 1E). This effect was mimicked by lactate and reversed by NaOx. BPA also upregulated SF1, CYP19A1, and ERβ mRNA levels; NaOx fully reversed SF1 and CYP19A1 expression, but only partially suppressed ERβ, while lactate replicated the BPA effect (Figure 1F). BPA increased extracellular lactate levels, reversed by NaOx and mimicked by lactate (Figure 1G). LDH activity followed a similar trend (Figure 1H).

### Transient BPA exposure induces persistent SF1 upregulation and oxidative stress; NaOx and AC45594 ameliorate the effects

**Figure 2:**
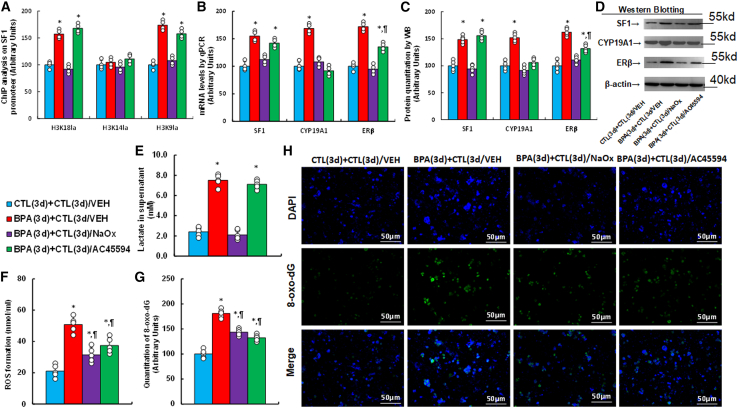
Transient BPA exposure causes persistent SF1 upregulation and oxidative stress, partially reversed by NaOx or AC45594 HESC were treated with CTL or 50 nM BPA for 3 days, followed by 3-day treatment with vehicle, NaOx (20 mM) or AC45594 (5 μM). (A) ChIP analysis of the SF1 promoter (*n* = 4). (B) SF1 mRNA (*n* = 4). (C) Protein quantification (*n* = 5). (D) Representative western blots. (E) Lactate levels (*n* = 5). (F) ROS generation (*n* = 5). (G) 8-oxo-dG quantification (*n* = 5). (H) Representative images of 8-oxo-dG staining. *n* means number of independent repeats. ∗, *p* < 0.05 vs. CTL(3d) + CTL(3d)/VEH; ¶, *p* < 0.05 vs. BPA(3d) + CTL(3d)/VEH. Data are presented as mean ± SD.

After 3 days of BPA treatment followed by 3-day of withdrawal, persistent histone lactylation at H3K18la and H3K9la was observed (Figure 2A). NaOx and SF1 inhibitor AC45594 both reversed this effect. BPA-induced expression of SF1, CYP19A1, and ERβ also persisted after BPA removal; NaOx reversed all, while AC45594 suppressed CYP19A1 and partially reduced ERβ but did not affect SF1 (Figure 2B). Protein expression mirrored mRNA patterns (Figures 2C, 2D, and S1A). BPA-induced lactate remained elevated post-withdrawal, reversed by NaOx but not by AC45594 (Figure 2E). ROS and 8-oxo-dG levels were also elevated post-exposure, and both inhibitors partially attenuated these effects (Figures 2F–2H). Similar trends were confirmed in HEECs for histone lactylation and gene expression (Figures S2A and S2B).

### Transient BPA exposure leads to sustained mitochondrial dysfunction ameliorated by NaOx and AC45594

**Figure 3:**
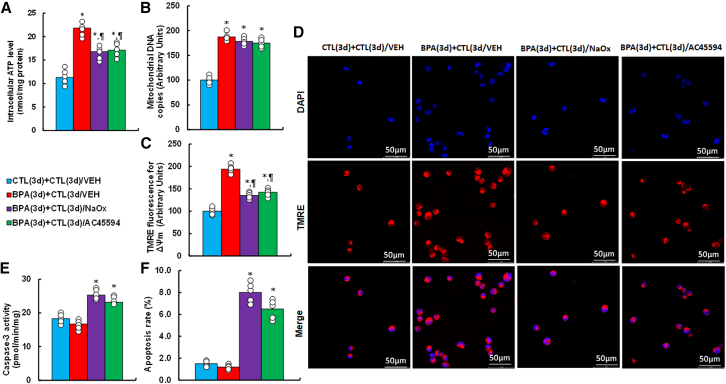
BPA exposure induces mitochondrial dysfunction, mitigated by NaOx or AC45594 HESC were treated with CTL or 50 nM BPA for 3 days, followed by 3-day treatment with vehicle, NaOx (20 mM), or AC45594 (5 μM). (A) Intracellular ATP. (B) Mitochondrial DNA copy number. (C) Mitochondrial membrane potential (ΔΨm). (D) Representative TMRE images. (E) Caspase-3 activity. (F) Apoptosis rate. *n* means number of independent repeats, *n* = 5. ∗, *p* < 0.05 vs. CTL(3d)+CTL(3d)/VEH; ¶, *p* < 0.05 vs. BPA(3d) + CTL(3d)/VEH. Data are presented as mean ± SD.

BPA exposure increased intracellular ATP, mtDNA copy number, and mitochondrial membrane potential (Δψm), effects that persisted 3 days after BPA removal (Figures 3A–3D). Both NaOx and AC45594 partly reversed elevated ATP and Δψm, but not mtDNA copy number. Caspase-3 activity and apoptosis were unchanged by BPA, but both increased significantly upon treatment with NaOx or AC45594 (Figures 3E and 3F).

### BPA-induced persistent cell proliferation is reversed by NaOx and AC45594

**Figure 4:**
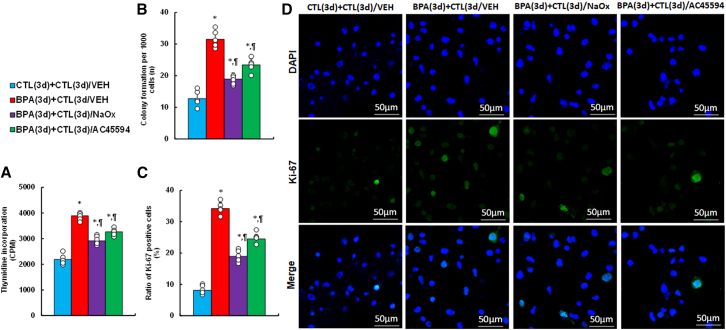
BPA exposure enhances cell proliferation, attenuated by NaOx or AC45594 HESC were treated with CTL or 50 nM BPA for 3 days, followed by 3-day treatment with vehicle, NaOx (20 mM), or AC45594 (5 μM). (A) Thymidine incorporation (CPM). (B) Colony formation. (C) Ki67-positive cell ratio. (D) Representative Ki67 images. *n* means number of independent repeats, *n* = 5. ∗, *p* < 0.05 vs. CTL(3d) + CTL(3d)/VEH; ¶, *p* < 0.05 vs. BPA(3d) + CTL(3d)/VEH. Data are presented as mean ± SD.

BPA treatment resulted in sustained cell proliferation, as shown by increased thymidine incorporation, colony formation, and Ki67-positive cells even after BPA withdrawal (Figures 4A–4D). These effects were partially reversed by either NaOx or AC45594.

### Postnatal treatment of either NaOx or AC45594 partly ameliorates prenatal BPA exposure-mediated oxidative stress and inflammation in circulation systems in female mouse offspring

**Figure 5:**
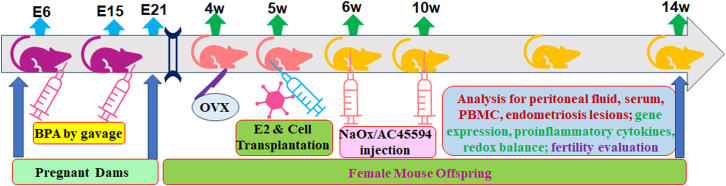
Schematic diagram of the *in vivo* mouse protocol Abbreviations: AC45594, SF1 inhibitor; BPA, bisphenol A; E, embryonic day; E2, estradiol; NaOx, sodium oxamate; OVX, ovariectomy; PBMCs, peripheral blood mononuclear cells; w, weeks.

**Figure 6:**
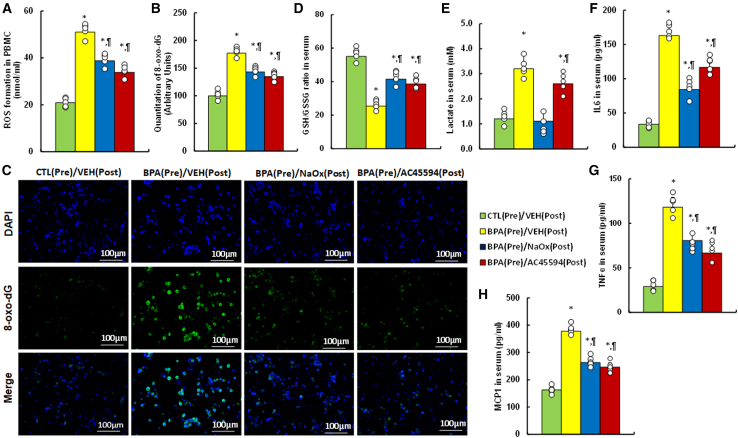
Postnatal NaOx or AC45594 treatment reduces oxidative stress and inflammation in female offspring prenatally exposed to BPA Female offspring prenatally exposed to BPA or CTL received postnatal treatment with vehicle, NaOx (250 mg/kg), or AC45594 (10 mg/kg) for 4 weeks. (A) ROS levels in PBMC. (B) 8-oxo-dG quantification in PBMC. (C) Representative images for (B). (D–H) Serum assays: (D) GSH/GSSG ratio, (E) lactate, (F) IL-6, (G) TNFα, (H) MCP1 (*n* = 5). *n* means number of animals, *n* = 5. ∗, *p* < 0.05 vs. CTL(Pre)/VEH(Post); ¶, *p* < 0.05 vs. BPA(Pre)/VEH(Post). Data are presented as mean ± SD.

We evaluated the potential effects of NaOx or AC45594 postnatal treatment on prenatal BPA exposure-mediated female mouse offspring (see mouse protocol in Figure 5). It showed that prenatal BPA exposure (BPA[Pre]/VEH[Post]) significantly increased the ROS formation (Figure 6A) and 8-oxo-dG formation (Figures 6B and 6C) in PBMC, and decreased GSH/GSSG ratio in serum (Figure 6D), compared to control group (CTL[Pre]/VEH[Post]); both NaOx and AC45594 treatment partly ameliorated those effects. Also, prenatal BPA exposure significantly increased the lactate in serum compared to control group; NaOx completely, while AC45594 partly, reversed the effects (Figure 6E). We finally determined the pro-inflammatory cytokines in serum and showed that prenatal BPA exposure significantly increased the cytokine levels of IL6 (Figure 6F), TNFα (Figure 6G) and MCP1 (Figure 6H) compared to control group; both NaOx and AC45594 partly reversed those effect. In addition, we determined peritoneal fluid levels of those cytokines, including IL6 (Figure S3A), TNFα (Figure S3B), and MCP1 (Figure S3C), and the similar secretion pattern was observed as of serum levels.

### Postnatal treatment with either NaOx or AC45594 partially ameliorated prenatal BPA-induced endometriosis development in female mouse offspring

**Figure 7:**
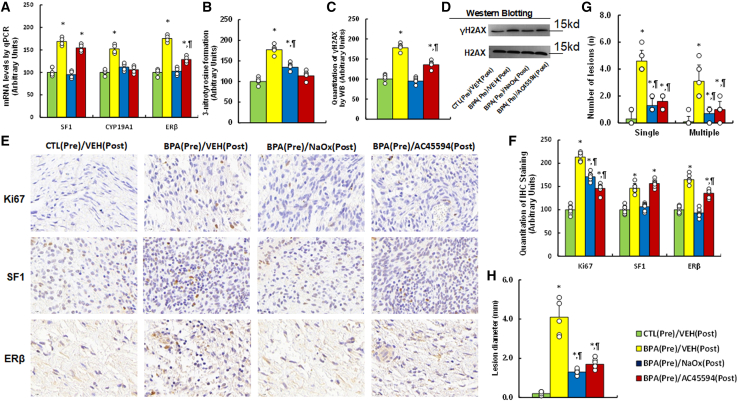
NaOx or AC45594 treatment mitigates prenatal BPA-induced endometriosis in female offspring Female offspring prenatally exposed to BPA or CTL received postnatal treatment with vehicle, NaOx (250 mg/kg), or AC45594 (10 mg/kg) for 4 weeks. (A) Gene expression by qPCR (*n* = 4). (B) 3-nitrotyrosine levels (*n* = 5). (C) γH2AX quantification (*n* = 5). (D) Representative blots. (E) IHC images for Ki67, SF1, ERβ. (F) IHC quantification (*n* = 9). (G) Lesion count; (H) lesion diameter (*n* = 9). *n* means number of animals. ∗, *p* < 0.05 vs. CTL(Pre)/VEH(Post); ¶, *p* < 0.05 vs. BPA(Pre)/VEH(Post). Data are presented as mean ± SD.

To evaluate their therapeutic effects, endometriotic tissues were collected for molecular and histological analyses. Histological examination confirmed that lesions displayed typical endometriosis features, including well-defined epithelial glands surrounded by proliferative stromal compartments, and BPA exposure was associated with increased cellular density and enhanced proliferative activity within both epithelial and stromal regions. Prenatal BPA exposure significantly upregulated the expression of SF1, CYP19A1, and ERβ. NaOx treatment completely reversed the BPA-induced increases in all three genes, whereas AC45594 fully reversed CYP19A1 expression, partially reduced ERβ expression, and had no significant effect on SF1 (Figure 7A). Assessment of oxidative stress markers showed that prenatal BPA exposure markedly increased 3-nitrotyrosine levels (Figure 7B) and γH2AX formation (Figures 7C, 7D, and S1B), indicating elevated nitrosative stress and DNA damage within lesion cells. Both NaOx and AC45594 treatments either partially or completely attenuated these effects. Immunohistochemical analysis further demonstrated that BPA exposure significantly increased Ki67, SF1, and ERβ staining, predominantly localized in glandular epithelial and adjacent stromal cells. NaOx treatment partially reduced Ki67 expression and fully reversed SF1 and ERβ expression, whereas AC45594 partially decreased all three markers (Figures 7E and 7F). Consistently, morphological evaluation showed that prenatal BPA exposure significantly increased both the number of lesions (single and multiple; Figure 7G) and lesion size (Figure 7H). Postnatal treatment with either NaOx or AC45594 partially mitigated these BPA-induced effects.

### Prenatal BPA exposure-mediated female mouse offspring have impaired fertility compared with control group

We evaluated the fertility and estrous cyclicity in those female mouse offspring and found that prenatal BPA exposure (Pre-BPA)-mediated female offspring had decreased pregnancy rate (Figure S4A), increased time to pregnancy (Figure S4B), decreased implantation sites (Figure S4C), litter size (Figure S4D) and pup viability (Figure S4E), compared to control group (Pre-CTL). Also, offspring from BPA treatment (Pre-BPA) had increased cycle length (Figure S4F) and days in estrus (Figure S4G), no difference on days in diestrus (Figure S4H), and decreased regular cycles (Figure S4I) and puberty onset (Figure S4J) compared to control group (Pre-CTL).

## Discussion

**Figure 8:**
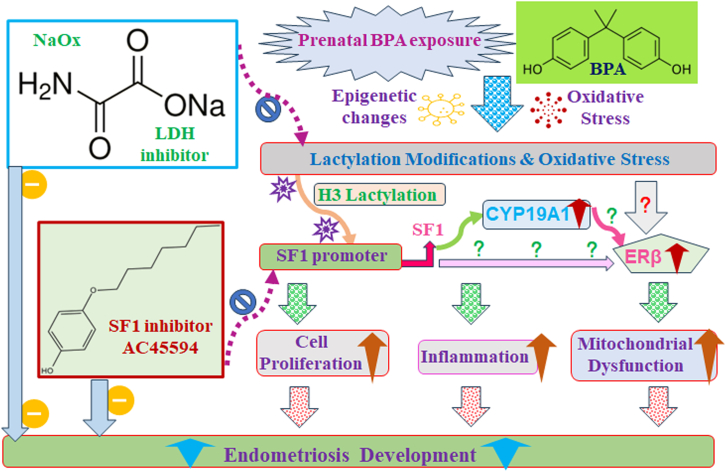
Schematic model: prenatal BPA exposure drives endometriosis development via histone lactylation and SF1 upregulation in female offspring Abbreviations: BPA, bisphenol A; ERβ, estrogen receptor β; H3, histone H3; LDH, lactate dehydrogenase; NaOx, sodium oxamate; SF1, steroidogenic factor-1.

Our results indicate that transient BPA exposure triggers persistent histone lactylation at the SF1 promoter, resulting in sustained upregulation of SF1 and its downstream targets, including CYP19A1 and ERβ. This cascade contributes to mitochondrial dysfunction, inflammation, and aberrant cell proliferation, hallmarks of endometriosis. Notably, pharmacological inhibition of lactate production with sodium oxamate (NaOx) or direct SF1 inhibition using AC45594 markedly mitigated the enhancement of endometriosis caused by prenatal BPA exposure in female mouse offspring, underscoring the therapeutic potential of targeting this pathway. We propose a model in which transient prenatal BPA exposure triggers oxidative stress and lactate accumulation, leading to histone lactylation on the SF1 promoter and subsequent upregulation of SF1, CYP19A1, and ERβ. These persistent epigenetic and metabolic changes promote mitochondrial dysfunction, inflammation, and cell proliferation, contributing to endometriosis development. Targeting this pathway with LDH inhibitor NaOx or SF1 inhibitor AC45594 offers a potential therapeutic approach (Figure 8).

### BPA promotes lesion formation via ERβ-dependent SF1 activation

Our findings reveal that SF1 plays a pivotal causal role in mediating the reproductive and endometriotic effects of prenatal BPA exposure. Pharmacological inhibition of SF1 by the selective antagonist AC45594 markedly attenuated lesion formation, proinflammatory cytokine expression (IL1β, TNFα), and oxidative stress in BPA-exposed offspring, demonstrating that SF1 activation is functionally required for disease manifestation. Mechanistically, BPA persistently upregulated SF1 mRNA and protein levels, accompanied by enhanced histone lactylation (H3K18la) at its promoter region, indicating an epigenetic mode of transcriptional activation. This observation aligns with the known ability of BPA to influence gene expression through estrogen receptor (ER)-mediated and chromatin-modifying mechanisms. In particular, the concurrent induction of ERβ and CYP19A1 suggests that BPA acts through an ERβ-SF1-aromatase regulatory axis, consistent with previous studies showing ER-dependent upregulation of SF1 and aromatase in endometrial cells.^17^^,^^28^ Collectively, these data establish a mechanistically coherent pathway in which BPA, acting via ERβ and epigenetic remodeling, enhances SF1 transcriptional activity to promote lesion formation, providing direct experimental evidence for SF1’s causative role in BPA-induced endometriosis-like phenotypes.

### Potential detrimental impact of BPA exposure during pregnancy on offspring

This study demonstrates that BPA exposure during pregnancy causes lasting reproductive abnormalities in female offspring, including increased susceptibility to endometriosis-like lesions and infertility. BPA disrupts developmental programming by inducing persistent histone lactylation and upregulating estrogen-related genes such as SF1, CYP19A1, and ERβ. These changes coincide with oxidative stress, mitochondrial dysfunction, inflammation, and enhanced cell proliferation-hallmarks of endometriosis. Therapeutic interventions targeting histone lactylation (NaOx) or SF1 (AC45594) partially reversed these effects, highlighting potential treatments. Susiarjo et al. showed that fetal BPA exposure disrupts meiotic prophase in oocytes, increasing aneuploid eggs via ERβ-mediated mechanisms, supporting our findings of SF1 and ERβ dysregulation linked to infertility.^29^ Caserta et al. reviewed 34 mouse studies revealing prenatal BPA impairs endometrial receptivity, reduces pregnancy rates, and alters estrogen and progesterone receptor (PGR) expression, reinforcing the connection between BPA exposure and compromised fertility.^30^ Our preliminary study found that PGR expression was undetectable in BPA-treated cells and lesions, indicating severe progesterone resistance. NaOx reduces histone lactylation and oxidative stress in disease models,^31^ while SF1 inhibitors like AC45594 suppress aberrant SF1 activity but require specificity improvements.^32^ These agents offer promising strategies to address BPA-related reproductive disorders.

### Oxidative stress as a driver of histone lactylation and SF1 upregulation

Our findings highlight oxidative stress as a central upstream mediator of BPA-induced epigenetic reprogramming. Transient BPA exposure caused long-lasting increases in both oxidative stress and histone lactylation, even after BPA withdrawal. This aligns with previous reports implicating oxidative stress in hyperglycemia-induced epigenetic changes.^33^^,^^34^ Reactive oxygen species (ROS) generated by BPA exposure may promote a metabolic shift toward aerobic glycolysis (the Warburg effect), thereby increasing intracellular lactate levels. Lactate, in turn, serves as a substrate for histone lactylation -a modification associated with transcriptional activation. Lactylation at regulatory regions of the SF1 gene may enhance its expression, further stimulating local estrogen production and inflammation, both of which are key contributors to endometriosis pathophysiology.^35^^,^^36^

### Lactate-mediated regulation of ERβ expression

We also observed that BPA exposure leads to significant upregulation of ERβ, a key mediator of endometrial proliferation and inflammation. Interestingly, LDH inhibition via NaOx effectively suppressed ERβ expression, whereas SF1 inhibition only partially reduced it. This suggests that ERβ upregulation is primarily driven by lactate-induced histone lactylation, independent of SF1. NaOx, by blocking lactate production, likely prevents lactylation-dependent activation of the ERβ promoter. Additionally, BPA’s estrogenic activity may indirectly increase ERβ expression by upregulating CYP19A1, a direct target of SF1, which encodes aromatase, an enzyme critical for local estrogen synthesis. Elevated estrogen may then further activate ERβ in a feedforward loop. These results support a model in which BPA modulates ERβ expression through both epigenetic and hormonal mechanisms, with histone lactylation acting as a central regulatory node.^36^^,^^37^^,^^38^

### Developmental origins of endometriosis and infertility

Our findings further support the concept of the developmental origins of endometriosis. Prenatal BPA exposure led to persistent oxidative stress, SF1 upregulation, mitochondrial dysfunction, and increased inflammation and cell proliferation in female offspring, culminating in endometriosis-like lesions and reduced fertility. BPA-induced ROS during critical windows of fetal development may irreversibly alter cellular metabolism and epigenetic landscapes, including histone lactylation, which in turn dysregulate genes involved in hormone signaling and immune responses.^35^^,^^36^^,^^37^ These developmental perturbations may program long-term susceptibility to reproductive disorders, consistent with epidemiological and experimental data linking prenatal endocrine disruption to adult reproductive disease.

### Limitations of the study

This study has several limitations. Although the prenatal BPA exposure mouse model recapitulates key features of endometriosis-like pathology and infertility, species-specific differences may limit direct translation to human disease. *In vitro* experiments using human endometrial stromal cells do not fully capture the complexity of the *in vivo* microenvironment, including immune, endocrine, and systemic metabolic interactions. The BPA exposure paradigm represents a defined dose and developmental window and may not reflect the variability and chronic low-dose exposures encountered in human populations. Moreover, while histone lactylation and SF1 activation were identified as central mechanisms, additional epigenetic modifications and signaling pathways contributing to disease progression were not examined. Finally, although NaOx and AC45594 showed partial therapeutic effects, their long-term safety, pharmacokinetics, and translational potential require further validation in clinically relevant models.

Our study demonstrates that prenatal BPA exposure enhances the susceptibility of female offspring to endometriotic lesion development. This effect is mediated through epigenetic and metabolic reprogramming, including increased histone lactylation and SF1 activation, which sustain estrogen-responsive gene networks, mitochondrial dysfunction, and chronic inflammation. Pharmacological inhibition of lactate production or SF1 activity partially mitigates these effects, highlighting a metabolic-epigenetic axis as a potential therapeutic target for environmentally influenced reproductive disorders.”

## Resource availability

### Lead contact

Further information and requests for resources and reagents should be directed to and will be fulfilled by the lead contact Dr. Paul Yao, (vasilis112@yahoo.com).

### Materials availability

This study did not generate new unique reagents.

### Data and code availability

• The information is accessible in both the article and supplemental information.
• Not Applicable
• The information is accessible in both the article and supplemental information.

## Acknowledgments

Authors are grateful to Mr. Jingtian Tang for his assistance with some biological assays and data analysis.

This study was kindly supported by Sun Yat-Sen Eighth Affiliated Hospital Clinical Research Project #: PY-2024-002 and Futian Healthcare Research Project #: FTWS039.

## Author contributions

L.Z. and P.Y. wrote the manuscript and supervised the entire study. L.H. and D.L. carried out some mouse experiments. X.L. and Y.F. conducted the remaining analysis.

## Declaration of interests

The authors declare no competing interests.

## STAR★Methods

### Key resources table

**Table:** 

REAGENT or RESOURCE	SOURCE	IDENTIFIER
**Antibodies**		

ERβ antibody	Cell Signaling Technology	Cat# 8644
SF1 antibody	Abcam	Cat# ab217317
Ki-67 antibody	Abcam	Cat# ab15580
γH2AX antibody	Cell Signaling Technology	Cat# 9718
β-actin antibody	Sigma-Aldrich	Cat# A5441

**Experimental models: Cell lines**		

Primary human endometrial epithelial cells (HEEC)	ZQXZ Bio (Shanghai, China)	Cat# PRI-H-00048
Primary human endometrial stromal cells (HESC)	ZQXZ Bio (Shanghai, China)	Cat# PRI-H-00098
Chemicals		
Bisphenol A (BPA)	Sigma-Aldrich	CAS: 80-05-7
17β-estradiol	Sigma-Aldrich	CAS: 50-28-2
Sodium oxamate (NaOx)	Sigma-Aldrich	CAS: 565-73-1
Sodium L-lactate	Sigma-Aldrich	CAS: 72-17-3
AC-45594 (SF-1 inhibitor)	Tocris Bioscience	Cat# 3043
Phenol red–free DMEM	Gibco	Cat# 11880-028
Charcoal/dextran-treated fetal bovine serum	Gibco	Cat# 12676029
CM-H2DCFDA ROS probe	Invitrogen	Cat# C6827
TMRE mitochondrial membrane potential probe	Invitrogen	Cat# T669
DAPI nuclear stain	Invitrogen	Cat# D1306
MTT reagent	Sigma-Aldrich	Cat# M5655
HEEC complete medium kit	ZQXZ Bio (Shanghai, China)	Cat# PCM-H-068
HESC complete medium kit	ZQXZ Bio (Shanghai, China)	Cat# PCM-H-120

**Critical commercial assays**		

3-Nitrotyrosine ELISA kit	Abcam	Cat# ab116691
Lactate assay kit	Nanjing Jiancheng Bioengineering Institute	Cat# A019-2-1
LDH activity assay kit	Nanjing Jiancheng Bioengineering Institute	Cat# A020-1-1
IL6 ELISA kit	R&D Systems	Cat# M6000B
TNFα ELISA kit	R&D Systems	Cat# MTA00B
MCP1 ELISA kit	R&D Systems	Cat# MJE00B
In Situ Cell Death Detection Kit (TUNEL)	Roche	Cat# 11684795910
ApoAlert Caspase-3 Activity Kit	Clontech	Cat# 630216

**Experimental models: Organisms/strains**		

Athymic nude mice (female)	Charles River Laboratories	Strain: Crl:NU(NCr)-Foxn1nu; RRID:IMSR_CRL:490

**Recombinant DNA**		

pGL3-Basic luciferase reporter vector	Promega	Cat# E1751
Renilla luciferase control vector (pRL-TK)	Promega	Cat# E2241

**Software and algorithms**		

ImageJ software	National Institutes of Health (NIH)	RRID:SCR_003070
Primer3 software	Primer3	RRID:SCR_003139

**Other**		

Dual-Luciferase Reporter Assay System	Promega	Cat# E1910
Confocal microscope (TCS SP8)	Leica Microsystems	Model TCS SP8

### Experimental model and study participant details

#### Cell lines and primary cell cultures

Primary human endometrial epithelial cells (HEEC; Cat# PRI-H-00048) and primary human endometrial stromal cells (HESC; Cat# PRI-H-00098) were obtained from ZQXZ Bio (Shanghai, China). Cells were cultured using HEEC complete medium kit (Cat# PCM-H-068) and HESC complete medium kit (Cat# PCM-H-120) (ZQXZ Bio), respectively, and maintained at 37°C in a humidified atmosphere with 5% CO_2_.

Cell identity was verified by immunostaining for cytokeratin-19 (CK19) in HEECs and vimentin in HESCs. Cells were used at passage 3 for all experiments. To extend proliferative capacity and improve transfection efficiency, cells were conditionally immortalized using a lentiviral vector encoding human telomerase reverse transcriptase (hTERT), as previously described.

To minimize background estrogenic activity, culture medium was replaced 24 h prior to treatment with phenol red–free DMEM (Gibco; Cat# 11880-028) supplemented with 10% charcoal/dextran-treated fetal bovine serum (Gibco; Cat# 12676029).

Cell authentication was performed based on cell type–specific marker expression as described above. Cells were routinely monitored for mycoplasma contamination using standard laboratory quality control procedures and were confirmed to be mycoplasma-free.

#### Experimental animals

Athymic nude mice (female; strain Crl:NU(NCr)-Foxn1nu; RRID:IMSR_CRL:490) were obtained from Charles River Laboratories. Mice were housed at 4–5 animals per cage under specific pathogen–free conditions with a 12 h light/12 h dark cycle, controlled temperature and humidity, and *ad libitum* access to food and water. BPA-free cages and water bottles, as well as a low-phytoestrogen diet, were used to minimize environmental estrogen exposure.

All animal procedures were conducted in accordance with the guidelines of the National Institutes of Health (NIH) for the care and use of laboratory animals and were approved by the Institutional Ethical Committee of the Eighth Affiliated Hospital of Sun Yat-Sen University (Approval No. #2024-283-01). All efforts were made to minimize animal suffering. Anesthesia was performed using isoflurane inhalation or ketamine (100 mg/kg) and xylazine (10 mg/kg) administered intraperitoneally.

For prenatal exposure studies, 8-week-old female mice were mated, and pregnant dams were randomly assigned to control or bisphenol A (BPA; CAS: 80-05-7; Sigma-Aldrich) exposure groups. BPA was administered at 50 μg/kg/day via oral gavage during gestational days 6–15. Female offspring were used for all subsequent experiments. Offspring were 4–8 weeks of age depending on the experimental protocol, including ovariectomy, hormone supplementation, cell transplantation, and pharmacological treatments.

##### Sex as a biological variable

All *in vivo* experiments were performed using female mice to model female reproductive physiology and endometriosis-related outcomes. Sex was therefore an inherent biological variable in this study, and no comparisons between sexes were performed.

### Method details

#### Cell culture and treatments

Primary human endometrial epithelial cells (HEEC; Cat# PRI-H-00048) and endometrial stromal cells (HESC; Cat# PRI-H-00098) were obtained from ZQXZ Bio (Shanghai, China). HEEC and HESC were maintained using the manufacturer-provided complete media systems (PCM-H-068 and PCM-H-120 kits, respectively). Cell lineage authenticity was verified by immunostaining for cytokeratin-19 in epithelial cells and vimentin in stromal cells. All experiments were performed using passage-3 cultures. To extend proliferative capacity and facilitate reporter transfection and *in vivo* implantation studies, cells were conditionally immortalized using a lentiviral vector expressing hTERT according to previously described procedures.^39^

Cells were grown at 37 °C under humidified conditions in an atmosphere supplemented with 5% CO2. To reduce background estrogenic effects, phenol red-free DMEM enriched with 10% fetal bovine serum that had been pretreated with charcoal/dextran (Gibco) was used for all chemical exposure experiments beginning 24 h prior to treatment.

Bisphenol A (BPA; ≥99% purity) was dissolved in ethanol to prepare a 10mM stock solution stored at −20°C protected from light. Working solutions were freshly prepared in phenol-red-free medium. Exposure concentrations ranged from 1 nM to 10 μM to encompass environmentally relevant and higher exposure levels reported to activate ERβ signaling and aromatase expression.^40^^,^^41^ For mechanistic studies, 100 nM and 1 μM were selected to represent low- and high-dose exposure. Vehicle controls contained 0.1% ethanol. Unless specified, cells were treated for 72 h, corresponding approximately to one complete cell cycle.

17β-estradiol (≥98% purity) was applied at 10nM as a positive estrogenic control. Sodium oxamate (NaOx) was used at 20mM to inhibit lactate dehydrogenase activity and suppress intracellular lactate accumulation and histone lactylation.^31^ Sodium L-lactate was added at 10 mM in designated rescue experiments. The SF-1 inhibitor AC-45594 was dissolved in DMSO to prepare stock solutions and applied at 1 μM final concentration, consistent with reported selective inhibition of NR5A1 transcriptional activity without cytotoxicity.^32^

Antibodies used for Western blotting, immunostaining, and ChIP targeted ERβ, SF1, Ki-67, histone lactylation marks, oxidative stress indicators, DNA damage markers, and loading controls, with species-matched IgG serving as negative controls.^24^ Nuclear and cytoplasmic protein fractions were prepared using commercial extraction reagents, and protein concentrations were determined using a Coomassie-based assay.

#### Plasmid construction

To generate human SF-1 promoter reporters, a genomic fragment spanning approximately a 2-kb region upstream of the transcription initiation site, together with exon 1 of NR5A1 (Ensembl transcript ENST00000373587.3) was amplified from human genomic DNA. The amplified fragment was inserted into the pGL3-basic luciferase reporter vector using standard restriction cloning. Sequential promoter deletion constructs were produced by PCR amplification and subcloning to evaluate regulatory regions controlling transcriptional activity.

#### Quantitative real-time PCR

Total RNA was isolated using a silica membrane-based extraction kit and reverse-transcribed into cDNA using a commercial reverse transcription system. Primers were designed using Primer3 software with optimal melting temperature and amplicon length criteria and validated for amplification efficiency. Quantitative real-time PCR was carried out with SYBR Green detection. Transcript abundance was determined using the 2ˆ−ΔΔCt approach, normalizing target gene expression to β-actin as the internal control.^42^^,^^43^

#### Cell viability and proliferation assays

Cell metabolic activity was evaluated using an MTT reduction assay. Following the indicated treatments, cells were exposed to MTT reagent, and the resulting formazan precipitates were subsequently dissolved prior to absorbance measurement using SDS-based extraction buffer prior to spectrophotometric measurement.^44^ DNA synthesis was assessed by incorporation of radiolabeled thymidine into cellular DNA followed by precipitation and scintillation counting.^43^^,^^45^ Anchorage-independent growth capacity was determined using a soft agar colony formation assay in which cells were suspended in agarose-containing medium layered over a solidified base agar. Colonies exceeding predefined size criteria were counted after extended incubation.^43^

#### Luciferase reporter assay

Cells were seeded into six-well plates and co-transfected with SF-1 promoter–driven luciferase constructs along with a Renilla luciferase vector to serve as an internal control. Following the indicated compound exposure, cells were harvested and lysed, and firefly and Renilla luciferase activities were quantified using a dual-luciferase assay system according to the manufacturer’s instructions.

#### Chromatin immunoprecipitation

ChIP assays were performed using approximately 2 × 10^6^ cells per reaction. Cells were crosslinked with formaldehyde, lysed, and chromatin was sheared by sonication. Immunoprecipitation was carried out using antibodies against transcription factors or histone lactylation marks, with IgG controls included. DNA was purified following reverse crosslinking and analyzed by quantitative PCR using promoter-specific primers.^42^^,^^43^

#### Western blot and immunofluorescence

Whole-cell extracts were generated using a lysis buffer containing detergents and a protease inhibitor cocktail. Equivalent amounts of protein were resolved by SDS–polyacrylamide gel electrophoresis and electrotransferred onto PVDF membranes. After incubation with specific primary antibodies, membranes were treated with fluorescence-labeled secondary antibodies, and protein bands were visualized with an infrared detection platform.^46^ For immunofluorescence analysis, cells were fixed and permeabilized prior to incubation with the indicated primary antibodies. After washing, appropriate fluorophore-tagged secondary antibodies were applied. Nuclear staining was performed with DAPI, and fluorescence signals were captured and quantified using dedicated image analysis software.

#### Measurement of oxidative stress

ROS formation was quantified using CM-H2DCFDA (10μM; Invitrogen) incubated at 37°C for 45min. Fluorescence was determined at 485/530nm and expressed in arbitrary units. 3-nitrotyrosine levels were assessed with an ELISA kit (Abcam, #ab116691). γH2AX was quantified via western blotting of nuclear fractions, with H2AX as the loading control.^47^

#### Mitochondrial function assessment

##### mtDNA copy number

Genomic and mitochondrial DNA were extracted using Qiagen kits. qPCR was performed for β-actin (nuclear control) and ATP6 (mitochondrial marker). Relative mtDNA copy number was calculated using the ΔΔCT method.^42^^,^^47^^,^^48^

##### Intracellular ATP

ATP levels were measured using a bioluminescence assay based on luciferase activity. A standard curve (10^-12^ to 10^-3^ M) was used for quantification. Values were standardized against overall protein levels and presented in units of nmol per mg.^42^^,^^47^^,^^48^

##### Membrane potential

Cells were loaded with 600nM TMRE for 20 minutes and imaged using confocal microscopy (Leica Microsystems, TCS SP8; excitation/emission: 548/573nm). Fluorescence intensities were analyzed in ImageJ from at least 10 cells per group.^48^

#### Apoptosis detection

TUNEL staining was conducted using the Roche *In Situ* Cell Death Detection Kit. Fluorescent TUNEL signals were captured via microscopy and further quantified via flow cytometry. The activity of caspase-3 was measured via the ApoAlert Kit (Clontech) using Ac-DEVD-AFC substrate, with fluorescence recorded at Ex/Em: 380/505 nm.^47^

#### Lactate measurement

Lactate levels in plasma or culture medium were measured using a colorimetric kit (#A019-2-1, Jiancheng, China) after centrifugation. The supernatants were placed on ice until analysis. A working solution containing lactate oxidase, peroxidase, and chromogenic substrates was prepared as directed by the manufacturer. In a 96-well microplate, 50μl of sample or standard was combined with 100μl of reagent and incubated for 30 min. Absorbance was determined at 530nm, and concentrations were calculated by reference to a standard curve constructed using known lactate standards.^24^

#### LDH enzymatic activity assay

To assess lactate dehydrogenase (LDH) activity, tissue or cell lysates were processed using an LDH assay kit (#A020-1-1, Jiancheng Bioengineering Institute). Lysates were prepared in chilled PBS and centrifuged for the supernatant. In a 200μl reaction volume, assay buffer, 0.2mM NADH, 2mM pyruvate, and 40-50μl of sample were mixed. The reaction was initiated by pyruvate addition, and the absorbance was monitored at 340 nm at 25°C every 30 seconds for up to 5 minutes. The rate of NADH oxidation was used to determine enzyme activity based on a molar extinction coefficient of 6.22 mM^-1^·cm^-1^. Values were normalized to protein concentration and reported as U/mg protein.

#### Cytokine quantification

Quantification of IL6, TNFα, and MCP1 in mouse serum or peritoneal fluid was performed using ELISA kits (IL-6: #M6000B, TNF-α: #MTA00B, MCP1: #MJE00B), from R&D Systems, in accordance with the protocols provided by the manufacturer.

#### Animal experiments

All animal procedures complied with NIH guidelines and institutional ethical regulations. Nude mice were maintained under controlled environmental conditions using BPA-free materials and a low-phytoestrogen diet.

#### Prenatal exposure protocol

Timed pregnancies were established by mating 8-week-old females with males. Confirmed pregnant dams were assigned into two groups (n=9): control (CTL) receiving 0.1% ethanol vehicle and BPA-treated receiving 50 μg/kg/day BPA administered orally via gavage starting on gestational day 6 and 15, respectively.

#### Postnatal Cell transplantation

After ovariectomy (OVX) surgery at 4-week old, female offspring were implanted subcutaneously with a 60-day 17β-estradiol (E2) release pellet (0.72mg) at 5-week old. Two days post-implantation, immortalized HEEC and HESC cells (2×10^6^ total) were mixed 1:1 in Matrigel (BD Biosciences), suspended in 150 μL volume, and injected intraperitoneally below the umbilicus. Care was taken to avoid organ damage. Mice were placed in a prone position to promote adherence.^49^

#### Postnatal chemical treatment

At 6 weeks, female offspring from Prenatal Exposure Protocol were allocated to four groups (n=9/group): CTL(Pre)/VEH(Post); BPA(Pre)/VEH(Post); BPA(Pre)/NaOx(Post): received intraperitoneal injections of NaOx (250 mg/kg in 0.9% NaCl) every other day for 4 weeks; BPA(Pre)/AC45594(Post): received intraperitoneal injections of AC45594 (10 mg/kg in 0.1% DMSO) every third day for 4 weeks.

#### Lesion evaluation

After treatment, mice were euthanized by CO_2_. Blood, peritoneal fluid, and PBMC were collected. Endometriotic lesions were identified, counted, and determined using calipers under a dissecting microscope. Lesions were categorized as single in the presence of one nodule, whereas two or more nodules were classified as multiple.^49^^,^^50^ PBMC was employed for gene expression analysis; serum for GSH/GSSG and lactate; peritoneal fluid for cytokine profiling. Some lesions were processed for RT-qPCR and fixed in 4% paraformaldehyde for histological and immunohistochemical staining.

#### Fertility assessment

Female offspring from control or BPA-exposed dams were monitored until 8 weeks of age. They were then paired with fertile males (n=10 per group) for 2 weeks. Mating was verified by detecting a copulatory plug. Evaluated parameters included time to pregnancy, pregnancy success rate, implantation site number (visualized using Chicago Blue dye on GD7), litter size, and pup viability on postnatal day 4. Vaginal smears were collected for 2 weeks prior to mating to assess estrous cyclicity, including cycle length, time in estrus/diestrus, percentage of regular cycles, and vaginal opening (VO) day.^29^

#### Immunohistochemistry (IHC)

Frozen lesion sections (10μm) were fixed sequentially with 2% paraformaldehyde (15min, RT) and methanol (10min, 4°C), permeabilized and incubated by primary antibodies (Ki67, SF1, ERβ at 40 μg/mL) for 2 hours. Visualization was achieved using HRP-linked secondary antibodies and DAB. Quantification was performed using ImageJ on 120 cells per group.^48^^,^^50^

### Quantification and statistical analysis

Statistical analyses were performed using SPSS software (version 22.0; IBM). All quantitative data are presented as mean ± standard deviation (SD) unless otherwise specified. Data were first assessed for normality using the Shapiro–Wilk test and for homogeneity of variance using Levene’s test. To minimize pseudo-replication, the litter was defined as the experimental unit where applicable. Differences among multiple groups were evaluated by one-way analysis of variance (ANOVA) followed by the Tukey–Kramer post hoc test, whereas comparisons between two groups were conducted using an unpaired two-tailed Student’s t-test. Categorical variables were analyzed using the chi-square (χ^2^) test. The exact sample size (n), its definition, and the statistical methods applied for each experiment are indicated in the corresponding figure legends and/or results section. For *in vitro* experiments, n represents independent biological replicates unless otherwise noted; for *in vivo* studies, n denotes the number of animals per group. Measures of central tendency and variability are defined in the figure legends. No data were excluded unless explicitly stated. Investigators were blinded to group allocation during outcome assessment and data quantification where applicable. A two-sided P value < 0.05 was considered statistically significant.^43^^,^^51^
