# Supplementary material for: Prenatal bisphenol A exposure reprograms SF1-lactylation pathways to promote endometriosis susceptibility
Source: iScience. 2026 Apr 4;29(5):115608. doi: 10.1016/j.isci.2026.115608 (PMC13098527; doi:10.1016/j.isci.2026.115608)
Supplement: Document S1. Figures S1–S4 and Table S1 [file mmc1.pdf]

iScience, Volume *29*

Supplemental information

Prenatal bisphenol A exposure reprograms SF1-lactylation pathways to promote endometriosis susceptibility

Xiaohan Liu, Yanxia Fu, Liuxuan Huang, Donghan Li, Paul Yao, and Liqin Zeng

Prenatal Bisphenol A Exposure Reprograms SF1-Lactylation Pathways to Promote Endometriosis Susceptibility

Supplementary Materials

FIGURE S1

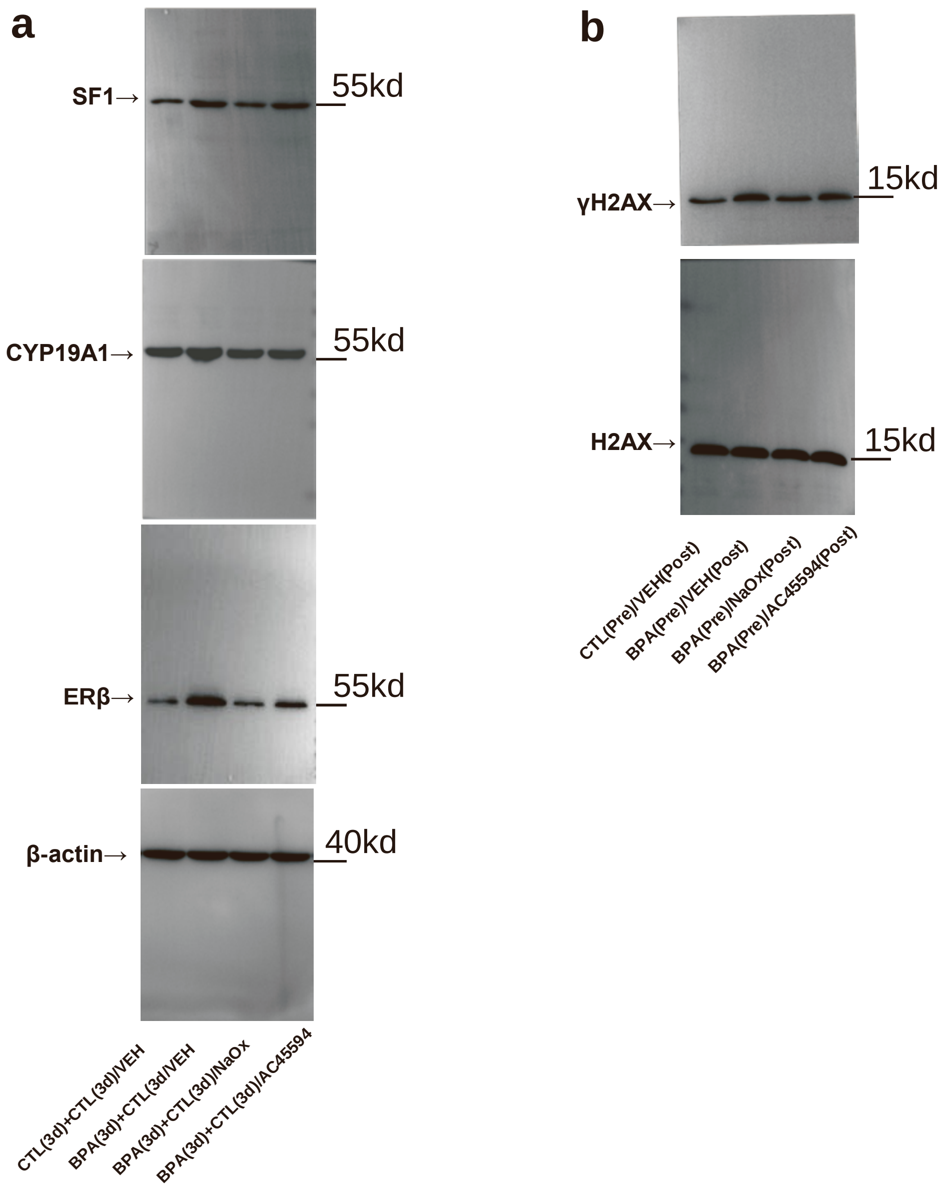

Figure S1. Representative pictures of full blots for Western Blotting. (a) Full blots for Figure 3h. (b) Full blots for Figure 7d.

FIGURE S2

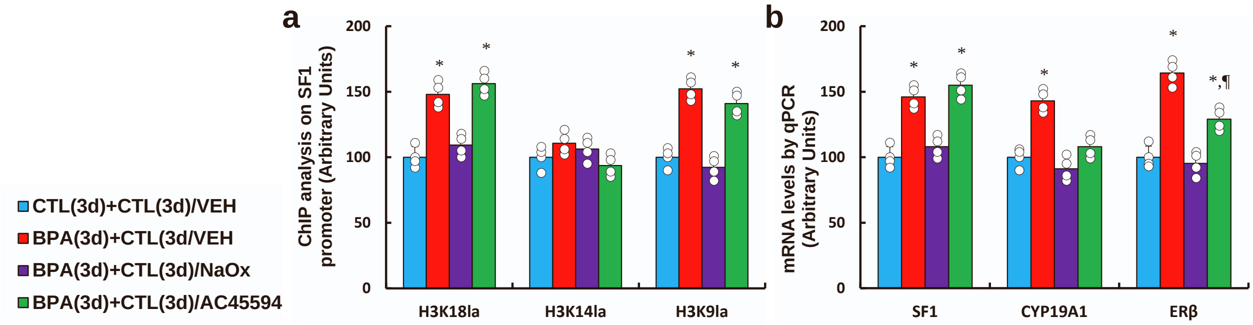

Figure S2. Transient BPA treatment induces persistent upregulation of SF1 and related genes through histone lactylation on the SF1 promoter in HEEC cells. HEEC cells were treated by either control (CTL) or 50nM of BPA for 3 days, then all the chemicals were removed, and treated by either vehicle (solvent only), 20mM of NaOx, or 5μM of SF1 inhibitor AC45594 for another 3 days, then cells were harvested for biological assays. (a) ChIP analysis on the SF1 promoter. (b) mRNA levels by qPCR. n means number of independent repeats, n=4. *, P<0.05 vs. CTL(3d)+CTL(3d)/VEH group; ¶, P<0.05, vs BPA(3d)+CTL(3d)/VEH group. Data are presented as mean ± SD.

FIGURE S3

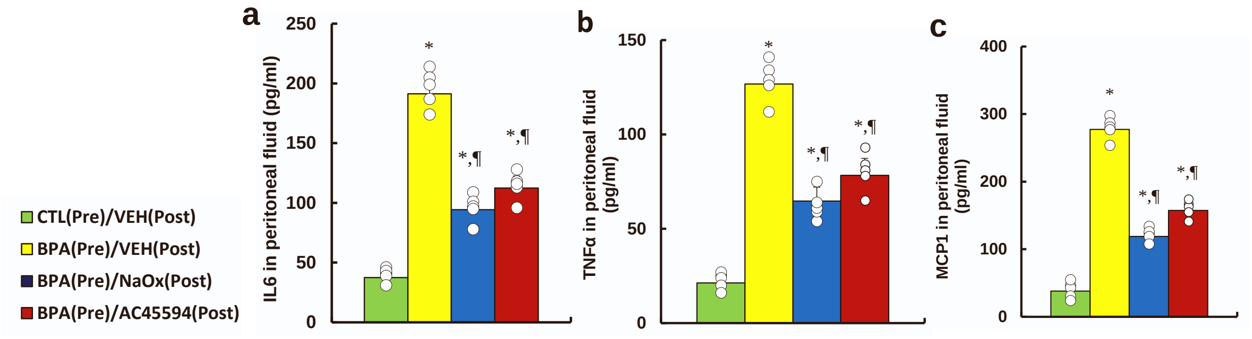

Figure S3. Postnatal treatment of either NaOx or AC45594 partly ameliorates prenatal BPA exposure-mediated proinflammatory cytokine release in peritoneal fluid in female mouse offspring. Prenatal exposure of either control (CTL) or BPA-mediated female mouse offspring received treatment of either solvent as vehicle control (VEH), 250mg/kg body weight of LDH inhibitor NaOx, or 10mg/kg body weight of SF1 inhibitor AC45594 for 4 weeks, and the peritoneal fluid was isolated for the analysis of proinflammatory cytokines of IL6 (a), TNFα (b) and MCP1 (c). n means number of animals, n=5. *, $P<0.05$ vs. CTL(Pre)/VEH(Post) group; ¶, $P<0.05$, vs BPA(Pre)/VEH(Post) group. Data are presented as mean ± SD.

FIGURE S4

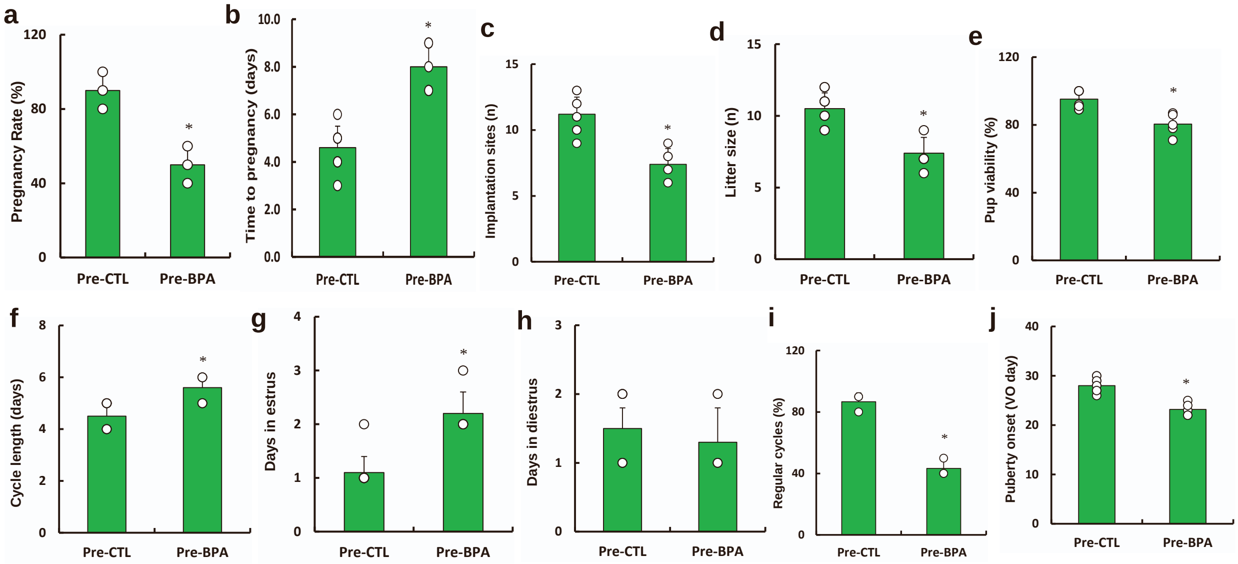

Figure S4. Prenatal BPA exposure-mediated female mouse offspring has impaired fertility compared with control group. Prenatal exposure of either control (CTL) or BPA-mediated female mouse offspring was used to evaluate the fertility. (a) pregnancy rate. (b) time to pregnancy. (c) implantation sites. (d) litter size. (e) pup viability. (f-j) estrous cyclicity was monitored for cycle length (f), days in estrus (g), days in diestrus (h), percentage of regular cycles (i) and puberty onset by vaginal opening (VO) day (j). n means number of animals, n=9. *, $P<0.05$ vs. Pre-CTL group. Data are presented as mean ± SD.

Table S1. Sequences of primers for the real time quantitative PCR (qPCR)

Gene	Species	Analysis	Forward primer (5'→3')	Reverse primer (5'→3')
β-actin	Human	mRNA	gatgcagaaggagatcactgc	atactcctgcttgctgatcca
SF1	Human	mRNA	ctgtgtccagtgtgtggtgac	gtcttgtcgattttgcagctc
CYP19A1	Human	mRNA	gactcgagtttttccccaaac	gtgtaacgaggatggctttca
ERβ	Human	mRNA	atgatgatgtccctgaccaag	acatcagccccatcattaaca
β-actin	Human	ChIP	tgtagcctgtacatcctccca	ggtctgcagtttgtacctgga
SF1	Human	ChIP	ccaggccttatgctagacacc	aggaagcactcctggattcat
β-actin	Mouse	mRNA	tcttgggtatggaatcctgtg	atctccttctgcatcctgtca
SF1	Mouse	mRNA	ctgtgtccagtgtgtggtgac	gtcttgtcgattttgcagctc
CYP19A1	Mouse	mRNA	tatgaacgatccgtcaaggac	ttctctttcgtcaggtctcca
ERβ	Mouse	mRNA	atgtgctatggccaacttctg	caagcttcctcttcagggtct
